# Effects of White Matter Microstructure on Phase and Susceptibility Maps

**DOI:** 10.1002/mrm.25189

**Published:** 2014-03-11

**Authors:** Samuel Wharton, Richard Bowtell

**Affiliations:** Sir Peter Mansfield Magnetic Resonance Centre, School of Physics and Astronomy, University of NottinghamUniversity Park, Nottingham, United Kingdom.

**Keywords:** Quantitative susceptibility mapping, phase imaging, microstructure, white matter, anisotropy, gradient echo MRI

## Abstract

**Purpose:**

To investigate the effects on quantitative susceptibility mapping (QSM) and susceptibility tensor imaging (STI) of the frequency variation produced by the microstructure of white matter (WM).

**Methods:**

The frequency offsets in a WM tissue sample that are not explained by the effect of bulk isotropic or anisotropic magnetic susceptibility, but rather result from the local microstructure, were characterized for the first time. QSM and STI were then applied to simulated frequency maps that were calculated using a digitized whole-brain, WM model formed from anatomical and diffusion tensor imaging data acquired from a volunteer. In this model, the magnitudes of the frequency contributions due to anisotropy and microstructure were derived from the results of the tissue experiments.

**Results:**

The simulations suggest that the frequency contribution of microstructure is much larger than that due to bulk effects of anisotropic magnetic susceptibility. In QSM, the microstructure contribution introduced artificial WM heterogeneity. For the STI processing, the microstructure contribution caused the susceptibility anisotropy to be significantly overestimated.

**Conclusion:**

Microstructure-related phase offsets in WM yield artifacts in the calculated susceptibility maps. If susceptibility mapping is to become a robust MRI technique, further research should be carried out to reduce the confounding effects of microstructure-related frequency contributions. **Magn Reson Med 73:1258–1269, 2015. © 2014 Wiley Periodicals, Inc.**

## INTRODUCTION

Phase images of the human brain acquired at high-field strengths using gradient echo (GE) MRI show exquisite tissue contrast 1–4. In most studies involving GE phase imaging, it is assumed that the dominant source of phase contrast is the variation in isotropic magnetic susceptibility across different tissues 5. This assumption has led to the development of a plethora of sophisticated techniques for inverting phase measurements to yield three-dimensional (3D) maps of the isotropic magnetic susceptibility 6–12. These “quantitative susceptibility mapping” (QSM) methods take advantage of the simple, Fourier relationship connecting the underlying distribution of isotropic magnetic susceptibility to the induced dipolar magnetic fields whose effect can be measured in phase images 13,14. Recently, however, several research groups have described additional mechanisms that could give rise to phase contrast in GE images: (i) exchange processes 15; (ii) nuclear magnetic resonance (NMR)-invisible microstructure 16; (iii) anisotropic magnetic susceptibility 17,18. Despite an increasing effort to quantify the contributions of each of these contrast mechanisms to phase measurements 19–23, the effect that these contributions have on susceptibility maps, calculated using QSM methods that assume isotropic magnetic susceptibility is the only cause of phase variation, has not been characterized. Only by understanding the impact of the additional phase contrast mechanisms on susceptibility mapping can the true value of QSM and other related methods be fully appreciated.

There are many potential applications of QSM due to the sensitivity of susceptibility maps to a range of endogenous biomarkers such as myelin 24,25, calcification 26, and iron content 8,27. A recent postmortem study showed that the measured isotropic susceptibility correlates well with iron content in deep gray matter (GM) structures 28, but the correlation between iron and susceptibility in white matter (WM) is weak. One explanation for the weak correlation in WM is the confounding contribution of other phase contrast mechanisms. In 2008, Zhong et al. 15 suggested that chemical exchange between water molecules and proteins could induce a local shift in the resonant frequency that affects the phase difference measured between GM and WM. In 2009, He and Yablonskiy 16 proposed that biased sampling of the magnetic field on the microscopic scale, due to the presence of NMR-invisible oriented microstructure, could lead to local frequency offsets that do not reflect the local magnetic susceptibility, while Marques et al. 29 showed that isotropic susceptibility could not fully explain WM phase contrast in an ex vivo murine brain. In 2010, Lee et al. 18 used measurements on fixed, postmortem tissue samples to show that the magnetic susceptibility of WM has a measurable anisotropy. Around the same time, Liu 17 showed that the full anisotropic magnetic susceptibility tensor can be reconstructed by solving the relevant inverse problem, provided that measurements of the field perturbation with the anisotropic susceptibility distribution oriented at a sufficient range of different orientations to the main magnetic field are available. This “susceptibility tensor imaging” (STI) method has been applied to phase images acquired at multiple orientations to the field from postmortem mouse brain 17, and more recently from the human brain in vivo 30.

Recent studies have suggested that the source of the anisotropic susceptibility of WM is the myelin sheath 19,30–32. This is composed of multiple lipid bilayers in which the lipid chains are radially oriented with respect to the approximately cylindrical sheath. The magnetic susceptibility in the myelin sheath is consequently described by a cylindrical symmetric tensor in which the principal axis is radially oriented. This geometry means that average magnetic susceptibility in a voxel containing multiple aligned nerve fibers is anisotropic because of the different average magnetic properties perpendicular and parallel to the fibers, but more interestingly the radial anisotropy in the myelin sheath also produces average frequency offsets of different polarity in the nerve sheath and lumen, whose magnitudes depends on the fiber geometry and orientation to the applied *B*_0_-field 19. As a result of the low water content of myelin and the rapid T2*-decay of the myelin water signal 33, the GE phase is more sensitive to the average frequency offsets inside the nerve lumens and in extraaxonal regions than to the offset in the myelin sheath. This gives rise to local frequency/phase offsets which are sensitive to the local WM microstructure 19,20,34.

The frequency contribution of microstructure was explored in a recent study by Luo et al. 22. These authors recorded frequency data from a rat optic nerve sample at multiple orientations to the main magnetic field and showed that there was a significant mismatch between measured frequency values and expected values based on a simple isotropic susceptibility model. Due to the experimental design utilized by Luo et al., it was not possible for them to estimate the anisotropic susceptibility of the WM sample. Here, we build upon this previous work and use a somewhat similar experimental setup to characterize the isotropic and anisotropic susceptibility and microstructural effects in a WM tissue sample.

In this study, we investigate the effects of susceptibility anisotropy and WM microstructure on QSM data. First, the isotropic and anisotropic magnetic susceptibility of a fresh sample of postmortem WM with known fiber orientation was quantified by comparing the measured external field variation with the sample at multiple orientations to the main magnetic field to field simulations. By subtracting the simulated WM frequency maps from the measured frequency maps, maps of the residual frequency offsets were formed. Inspection of the fiber-orientation dependency of these residual WM frequency offsets allowed the microstructure contribution to be characterized and separated from the unknown exchange contribution. Simulations were then carried out to investigate the effect of frequency offsets due to anisotropy and microstructure on QSM-based calculations of isotropic magnetic susceptibility. In addition, the artifacts caused by microstructure in STI processing were also investigated.

## METHODS

### White Matter Sample Preparation

Optic nerve forms an ideal structure for investigating phase offsets due to WM 22 because it forms a compact cylindrical structure containing many nerve fibers that are aligned with the axis of the optic nerve. Here, a section of optic nerve of around 0.4-cm diameter was harvested from a recently euthanized 60 kg pig and trimmed to a length of approximately 2 cm. The nerve section was embedded in agar (1.5% agarose gel made from 1% saline solution) contained in a Perspex sphere of 10-cm diameter. Imaging was carried out about 4 hours after the sample had been harvested.

### Image Acquisition

Images were acquired on a Philips Achieva 7T scanner (Philips Medical Systems, Best, the Netherlands) using a 32-channel receiver head coil. A dual-echo, 3D, spoiled GE sequence was used to image the spherical tissue phantom with parameters: repetition time (TR) = 28 ms; echo time (TE)_1_ = 7 ms; TE_2_ = 20 ms; field of view (FOV) = 112×112 ×70 mm^3^; flip-angle = 11^o^; scan time = 15 min; isotropic resolution = 0.5 mm; unipolar readout gradients. The tissue phantom was imaged with the optic nerve section oriented at 10 different angles to the *B*_0_-field, This resulted in 10 data sets in which the angle,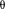, between the nerve axis and *B*_0,_varied between 0° and 90° in approximately even steps of 10°. The total scanning time was approximately 200 min. The first two image data sets were acquired with the nerve parallel (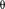
= 0) and then perpendicular (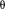
= 90°) to *B_0_,* while the order of subsequent acquisition was randomized in order to reduce any systematic errors due to tissue degradation over the course of the experiment.

A single healthy human subject of 28 years of age was also imaged at 7T (with informed consent and local ethical approval) using the same GE sequence as was used in the phantom experiments (see parameters above), but with a coarser isotropic resolution of 1 mm and at a single orientation. In addition, diffusion tensor imaging (DTI) data was acquired (sequence parameters: TE = 57 ms; TR = 8.6 s; FOV = 224 × 224 × 104 mm^3^, in-plane resolution = 2 mm; slice thickness = 2 mm; scan time = 7 min; 32 diffusion gradient directions, b-value = 1000 s mm^-2^) and T1-weighted anatomical images (sequence parameters: TE = 3.7 ms; TR = 8 ms; inversion time [TI] = 960 ms; long-TR = 2.8s; turbo field echo (TFE) factor = 205; flip angle = 8^o^; FOV = 256 × 256 × 160 mm^3^; isotropic resolution = 1 mm) of the same subject were acquired at 3T.

### Image Processing

The phase data from each echo were unwrapped using a fast 3D method 35. Frequency maps were formed by taking the difference of the unwrapped phase values associated with the first and second echoes and then scaling the result by the TE-difference (ΔTE =13ms). Similarly,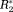 maps were formed from the dual-echo data by dividing the difference in the natural logarithm of the magnitude data associated with each TE by ΔTE. The magnitude images associated with the second echo of each of the 10 different data sets were coregistered using a rigid body transform in FMRIB's Linear Image Registry Tool 36 to a common sample space, in which the nerve fibers in the optic nerve section were aligned with the *z*-axis. The resulting rotation matrices were then applied to the associated frequency and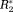 maps. The frequency maps were spatially filtered to remove unwanted background fields using the sophisticated harmonic artifact reduction for phase data (SHARP) method 8. The DTI data were processed using FSL dtifit 37 to yield maps of the fractional anisotropy (FA), and of the eigenvectors describing the fiber orientations.

### Calculation of Susceptibility From External Field

In standard susceptometry experiments, the known form of the external field perturbation due to a sample of specific geometry (e.g., an infinite cylinder perpendicular to *B*_0_) is compared to measured field data in order to calculate the relative isotropic magnetic susceptibility offset of the sample relative to the reference medium in which it is embedded 38. As shown by Lee et al. 18, this methodology can be extended to allow for calculation of the anisotropic magnetic susceptibility of a small fixed WM tissue sample. This approach is advantageous because by focusing on the external field perturbation it eliminates any sensitivity to exchange or microstructure, which only produce signal changes within the sample. In this study, we adopt a similar approach for calculating susceptibility based on measurement of the field variation induced outside the fresh tissue sample.

The simulated frequency map,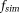, due to a sample embedded in a reference medium can be written as

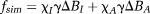
[1]where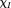 and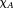 are scalar values describing the isotropic and anisotropic components of a cylindrically symmetric susceptibility tensor 19,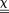, representing the magnetic properties of the sample relative to the surrounding medium such that

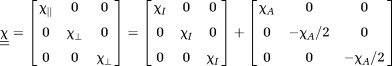
[2]where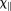 and
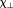
are the magnetic susceptibility of the sample parallel and perpendicular to the principal axis of the cylindrically symmetric susceptibility tensor.
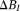
and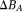 in Equation [1] represent the field perturbations per unit of isotropic and anisotropic magnetic susceptibility, and are given by:

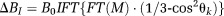
[3]

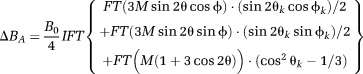
[4]

Here,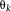 and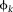 are spherical polar coordinates in k-space, with the *z*-axis aligned with the direction of *B_0_*,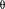, and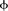 are spherical polar coordinates describing the direction of the principal axis of the susceptibility tensor (defined as the *x*-axis in Eq. [2]) relative to the *B*_0_ direction and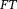 (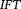) denotes a 3D (inverse) Fourier transform. The shape of the sample is represented by the digitized mask function, *M*, which takes a value of 1 in voxels lying within the sample and a value of 0 in voxels in the surrounding reference medium. Equation [3] is the standard Fourier representation of the field perturbation due to a 3D distribution of isotropic magnetic susceptibility 13,14. The derivation of the Fourier expression in Eq. [4], which can be used to calculate the field due to a zero-trace, cylindrically symmetric susceptibility tensor is described in our previous work 19,39. For this approach to be valid, the sample must have homogenous magnetic properties: That is, the values of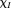 and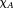, as well as the direction of the principal axis of the susceptibility tensor, must be uniform over the entire sample. The sample used in this study should satisfy these conditions because the direction of the nerve fibers, and the extent to which these nerve fibers are myelinated, is expected to be fairly uniform over a short section of optic nerve.

**Figure 1:**
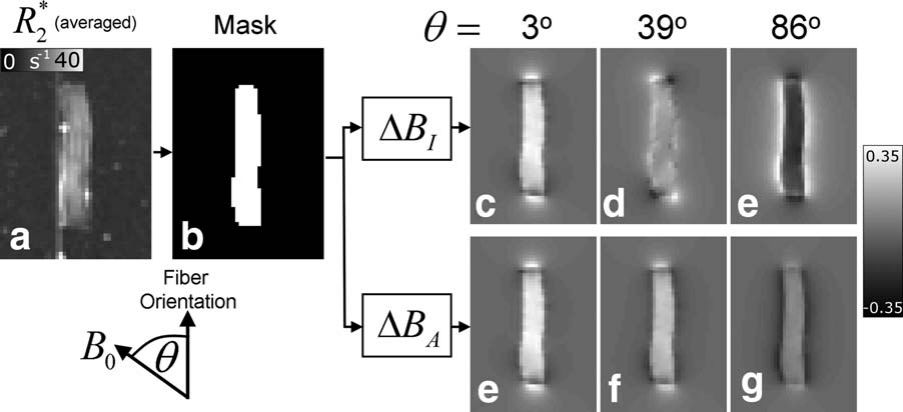
Steps followed in generating simulated field maps. The average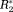 map (average over all 10 sample orientations) (a) was used to produce a mask of the optic nerve sample (b). Maps of the field perturbation due to the isotropic magnetic susceptibility (c–e) and anisotropic magnetic susceptibility (f–h) of the optic nerve sample were simulated for 10 different sampling orientations by applying Eqs. [3] and [4] to the optic nerve mask. Simulated field maps from three different sample orientations are shown.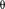 is the angle between *B*_0_ and the direction of the fibers in the sample.

Figure 1 shows the steps used to simulate the frequency map due to the optic nerve sample. A 3D mask of the WM sample (see Fig. 1b),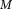, was drawn based on a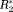 map (Fig. 1a) formed by averaging the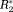 data from all sampling orientations. By substituting the sample mask into Eq. [3] and Eq. [4], the isotropic and anisotropic field perturbations maps were separately simulated for each of the 10 sampling orientations. As part of these calculations, the spherical polar coordinates in Eq. [3] and Eq. [4] had to be recalculated based on the direction of *B*_0_ for each sampling orientation. Representative slices of the simulated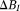 and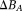 field maps are shown in Figure 1c to 1h for three different orientations. Least-squares fitting of the simulated frequency values in the region outside the mask to experimental measurements for all sample orientations was then used to estimate the values of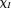 and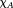 in the WM sample. The simulated frequency maps were spatially filtered using SHARP in an identical manner to the measured frequency data before the fitting was carried out. Only voxels external to the sample mask were included to avoid the confounding local effects of exchange and microstructure. The external region was defined by dilating the sample mask by 8 voxels in all dimensions followed by subtraction of the original sample mask dilated by 1 voxel. Also, to avoid including erroneous field values due to the presence of air-bubbles in the agarose gel, only voxels with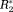 less than 15 s^−1^ were included in the fitting procedure, which was carried out using the “lscov” function in MATLAB 7.5.0 (MathWorks, MA, U.S.A.). This utilizes matrix inversion based on orthogonal decomposition. As this was a matrix inversion-based procedure, it was not necessary to define a fitting range, and the fitted values should yield a global minimum in the least-squares residual.

### Measuring residual frequency values

By fixing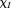 and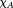 to the best-fitting values calculated from the fit to the external field and subtracting the resulting simulated frequency maps from the measured frequency maps, a map of the residual frequency offset,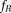, was formed at each sampling orientation. The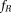-maps represent the measured frequency offsets that are poorly explained by the bulk isotropic and anisotropic susceptibility effects described by Eqs. [3] and [4]; therefore, they are likely to be dominated by contributions from the exchange and microstructure mechanisms local to the WM sample. To investigate this hypothesis, the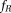 offset inside the optic nerve sample was measured by averaging over an ROI defined by eroding the mask,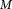, by 1 voxel. This was repeated for all sampling orientations to yield 10 average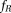 measurements.

The residual frequency,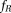, is expected to vary with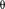 as:


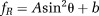
[5]

where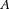 characterizes the amplitude of the variation in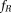 on rotating the sample, and
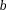
is a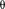-independent offset. The form of Eq. [5] is based on the assumption that the amplitude of the microscopic field variations due to myelinated nerve fibers, which underlie the microstructure contrast mechanism, varies as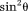 16,19,31,34. The measured *f_R_*-values were fitted to Eq. [5] to yield estimates of *A* and *b*. As exchange processes are insensitive to fiber orientation,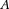 is expected to be solely dependent on microstructure effects. In contrast,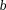 is likely to contain contributions from both exchange and microstructure 19.

### Simulating whole brain frequency maps

To investigate the effect of frequency offsets due to microstructure on QSM and STI, the frequency variations produced by a digitized model of the human brain were calculated. To generate the model, FMRIB's Automated Segmentation Tool 40 was first used to segment the T1-weighted anatomical brain image acquired from a single healthy subject, yielding digitized masks of WM and non-WM tissue (cerebrospinal fluid [CSF] and GM). As the focus of this study is the effect of WM microstructure on QSM and STI, the values of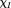 and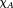 in CSF and cortical GM were set to zero. The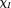 and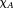 values used in the WM regions were based on the results of the optic nerve experiments. The estimates of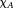 and the fitted microstructure-related amplitude parameter,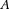, are absolute measures and are likely to manifest in human WM with similar values to those measured in the porcine optic nerve sample. However, the estimates of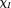 and the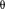-independent residual frequency offset,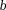, are relative measures that depend on the isotropic magnetic susceptibility and any exchange-related frequency offset in the media surrounding the WM. For the tissue phantom, the reference medium is agarose gel, but in the human brain, WM is mostly surrounded by GM. As there is insufficient data in the literature to form a robust estimate of the exchange-based frequency offset in GM relative to WM, a single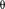-independent residual frequency offset was not included in the simulations. In contrast, sufficient literature now exists to suggest that WM has a diamagnetic isotropic susceptibility offset of the order of −0.05 ppm relative to GM 8,19,24. The simulations were, therefore, carried out with the value of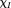 in WM set to −0.05 ppm relative to that in non-WM.

In addition to WM and cortical GM, iron-rich deep GM structures in the basal ganglia region can make large contributions to frequency maps acquired in vivo 4,5,7,8,10,11. In an effort to simulate these additional frequency offsets, masks of several deep GM regions were hand-drawn on the magnitude data associated with the second echo of the GE data set to yield 3D binary masks that could be populated with susceptibility values. The chosen structures were the globus pallidus (GP), putamen (PU), caudate nucleus (CN), thalamus (TH), and pulvinar part of the thalamus (PV). For each region, the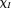 value relative to the surrounding WM was based on recent literature 7,8,41: GP = 0.15 ppm, PU = 0.05 ppm; CN = 0.05 ppm; TH = 0.00 ppm; PV = 0.03 ppm. Only isotropic susceptibility effects were considered because these iron-rich structures are not thought to exhibit strong anisotropy- or microstructure-related effects.

The frequency variation, *f_I_*, due to the isotropic susceptibility of the WM and deep GM structures in the brain model, was calculated using Eq. [3], with the WM and deep GM masks, multiplied by their respective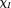-values, taking the place of *M*. To calculate the frequency variation due to the anisotropic susceptibility of WM, it is necessary to know the average fiber direction in each voxel, which dictates the direction of the principal axis of the susceptibility tensor. This information was derived from the DTI data and then used to evaluate the values of *θ* and *φ*, which characterize the average fiber orientation relative to the *B_0_-*field in each voxel. These values were fed into a modified version of Eq. [4], in which the mask *M* was replaced by the WM mask multiplied by a spatially varying value of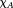 in order to calculate the frequency variation, *f_A_*, produced by the anisotropic susceptibility of the WM in the brain model. We allowed the value of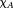 to vary in order to reflect the variation of the coherence of fiber orientations in voxels in different WM regions. For example, the fiber orientations are expected to be fairly uniform in a voxel in the corpus callosum (CC), but are likely to have a greater dispersion in voxels located in cortical white matter. As the magnitude of the anisotropy of the susceptibility tensor in a WM voxel is expected to be linked to the dispersion of fiber orientations, we modulated the magnitude of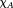 used in the simulations based on the FA measured from the DTI data. The FA values were normalized by dividing by a scaling factor 0.59 based on a literature value for the in vivo FA of the human optic nerve tissue 42. To simplify the simulations,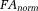 was set to zero in the segmented deep GM structures. The resulting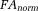 values were multiplied by the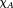 value estimated from the optic nerve experiments to yield a spatially varying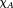 map, in which the local magnitude depends on the local FA estimated from the DTI experiment.

A spatially varying microstructure-related frequency offset,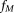, was also calculated using


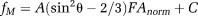
[6]

where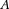 is the amplitude calculated from the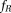-values measured in the optic nerve,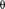 is the angle between *B*_0_ and the fiber orientation, which was derived from the DTI data, and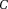 is a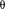-independent frequency offset. In a similar manner to the formation of the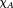-map,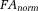 is included in Eq. [6] to generate a more realistic distribution of the magnitude of the microstructure offset. The factor of −2/3 was included in Eq. [6] to ensure that the average of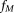 over a random distribution of fiber orientations is equal to zero. By defining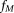 in this manner, the average effect of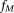 is more easily separable from the unknown,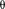-independent frequency offset represented by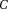.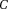 is included in Eq. [6] to model local exchange-induced,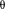-independent frequency offsets in WM 15, as well as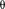-independent WM frequency shifts due to microstructure 19,20,23,34. Previous work by our group 19 and others 15,21,43 has suggested that there are exchange-related frequency offsets on the order of 0.01ppm in WM relative to GM, which at 7T corresponds to a frequency offset of ≈3Hz. To investigate the effects of including a range of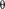-independent WM-GM offsets, frequency maps were simulated with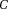 set to −3Hz, 0 Hz, and +3Hz. For consistency with the data from the in vivo experiments, where no frequency information is available outside of the brain, the simulated frequency maps were masked, using the mask produced by applying FMRIB's Brain Extraction Tool 44 to the T1-weighted anatomical data.

### Investigating Artifacts in QSM

In this study, QSM was carried out using the thresholded k-space division (TKD) method 9,45. This method was chosen due to its speed and simplicity. To assess the effect of the frequency offsets due to anisotropy and microstructure on the calculated susceptibility maps, TKD-based QSM was applied to three different frequency maps: (i) a frequency map due to purely isotropic susceptibility offsets,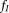; (ii) a frequency map containing isotropic and anisotropic contributions,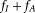; and (iii) a frequency map containing susceptibility and microstructure related contributions,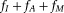. In these simulations,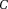 (in Eq. [6]) was set to zero (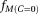). The TKD kernel threshold was set equal to 0.07, which corresponds to a truncation value of 14 in the reciprocal units proposed by Shmueli et al. 9. This value was chosen through trial and error as a good compromise between contrast and artefact-related noise 9. In an additional experiment, the fully relaxed TKD kernel proposed in recent work 45 was also applied to the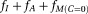 data set. As part of the TKD processing, a global correction factor was applied to compensate for the underestimation of susceptibility values due to kernel truncation 45. Difference from truth (DFT) maps were formed by subtracting the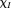 map (including WM and deep GM offsets) used in the simulations from the calculated susceptibility maps.

### Investigating Artifacts in Susceptibility Tensor Imaging

The effects of microstructure-related frequency offsets on STI were also investigated.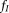,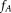, and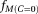 data were simulated, as described above, but for 16 different orientations of *B*_0_ with respect to the sample. These directions were chosen so that the tips of the *B_0_* vectors approximately were spread evenly over a hemispherical surface. This is equivalent to rotating the sample in the field of the scanner in order to properly condition the ill-posed inversion problem underpinning STI 17. In a similar manner to the QSM experiments, STI was separately applied to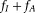 data and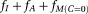 data. The STI data processing was carried out according to Liu 17, with a limit of 30 iterations imposed upon the conjugate gradient minimization algorithm. All QSM and STI processing was carried out in MATLAB using a 64-bit Linux system with a 2 GHz dual core AMD processor and 8 GB of RAM. The eigenvalues, (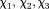), and eigenvectors of the resulting susceptibility tensors were calculated at each voxel 17. The reconstructed isotropic and anisotropic susceptibility values are then given by


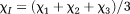
[7]


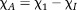
[8]

where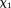 is the eigenvalue with the largest difference from the mean value (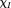). The eigenvector,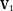, associated with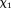, represents the estimated fiber direction. DFT maps were formed by subtracting the true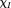 and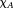 maps from the reconstructed susceptibility maps based on the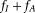 and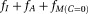 data. DFT maps were also formed for the reconstructed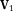 data by voxelwise calculation of the magnitude of the angle between the reconstructed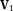 vector and the direction of the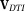 vector, used to simulate the fiber orientation.

## RESULTS

**Figure 2:**
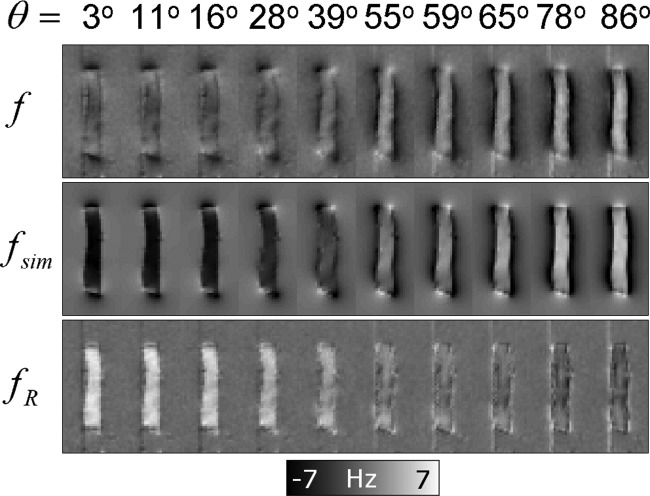
Results of the tissue phantom experiments. Coregistered frequency maps (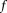) are shown for all 10 fiber orientations. The dipolar nature of the external field perturbation due to the optic nerve section and its rotation as the direction of *B*_0_ changes is evident from the measured frequency maps. The corresponding simulated frequency maps (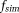), based on a best fit to the external frequency variations in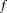, are also shown. The residual frequency maps (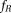) are generated by subtracting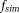 from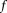 at each orientation. The residual frequency offsets in the agarose gel surrounding the sample are small, suggesting that the measured external field variations are well characterized by the simulation. Also, a subtle line can be seen in the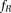 maps showing the layer of gel that was allowed to cool and harden to support the sample.

**Figure 3:**
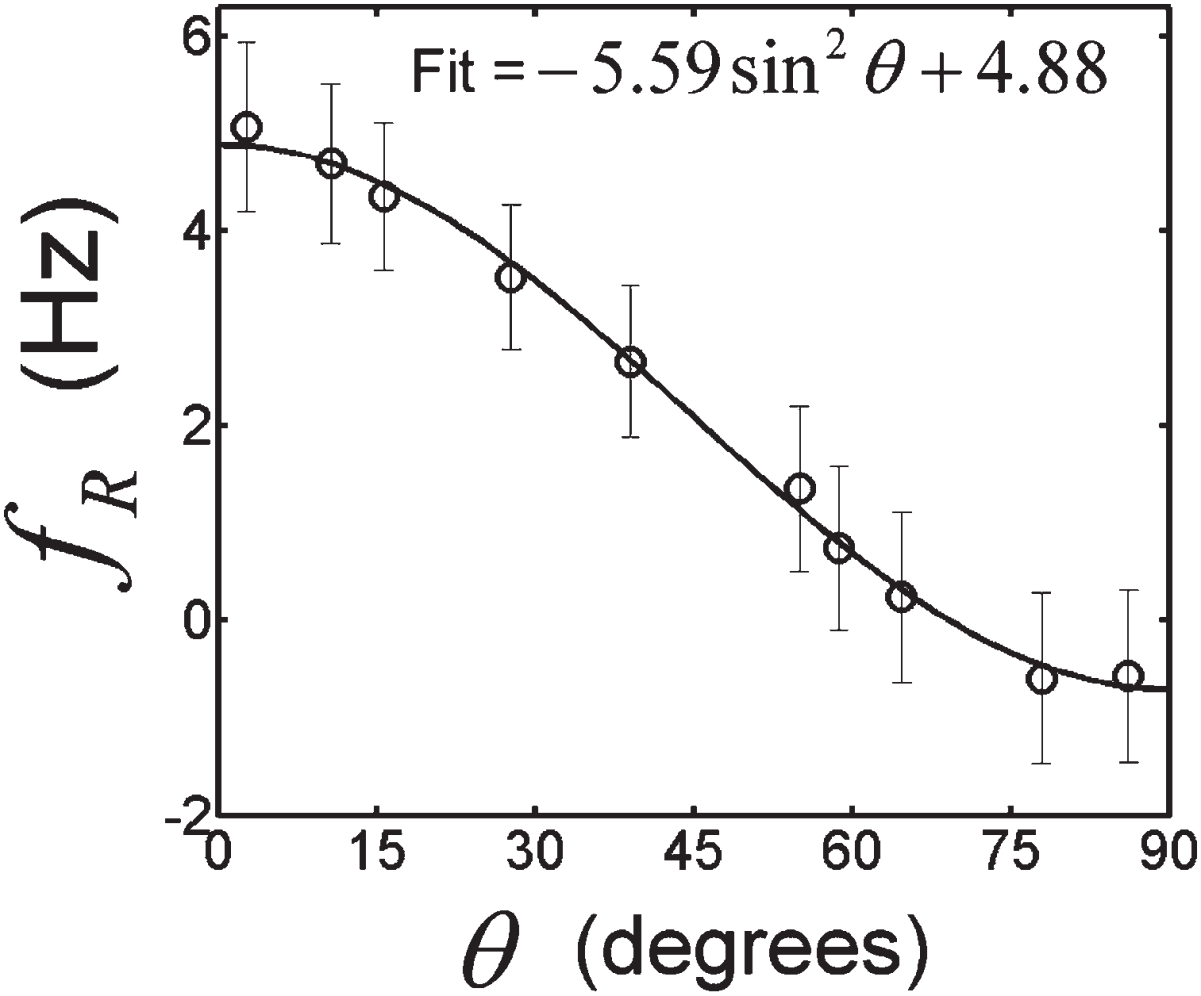
Plot of the average residual frequency (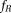) values inside the optic nerve against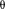. The error bars represent the pooled standard deviations and the solid line is the result of least squares fitting the curve in Eq. [5] to the measured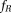-values.

Figure 2 shows the results of the tissue phantom experiments. The best-fitting values for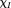 and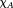, based on the external frequency variation in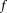 (1st row in Fig. 2), were found to be −0.08152 ± 0.00006 ppm and 0.01128 ± 0.00009 ppm, where the quoted errors are the estimated standard errors in the least-squares fitting procedure and take into account the large number of voxels that were sampled. The correlation between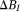 and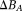 was fairly low (R = −0.318), suggesting that the fitting procedure was reasonably robust. Maps of the simulated frequency,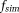, generated by substituting the fitted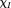 and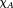 values into Eq. [1] are also shown in Figure 2 (2nd row), along with the maps of the variation of the residual frequency offset,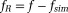 (3rd row in Fig. 2). The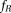 values inside the optic nerve are significant and positive for small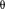 values, decreasing in magnitude as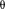 increases toward 90°. Figure 3 shows the variation with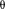 of the average value of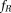 inside the optic nerve. The curve described in Eq. [5] provides an excellent fit to the measured, average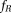-values, yielding coefficient values of *A=* −5.59 ± 0.12 Hz and *b =*4.88 ± 0.07 Hz. The fit was also carried out using only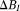 (i.e., ignoring anisotropy), which yielded a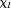 value of −0.083698 ± 0.00006 ppm and also produced average residual frequency offsets that were well modeled by Eq. [5] with coefficient values *A=* −6.53 ± 0.12 Hz and *b =* 5.97 ± 0.07 Hz. The results of an F-test suggested that, after taking into account the increased number of fitting parameters, the incorporation of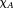 significantly improved the fit (*P* < 0.001). The importance of including a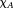 component is further demonstrated in Suppl. Figure 1, which shows a comparison of the residual frequency offsets in the external agarose compartment for the two different forward models.

**Figure 4:**
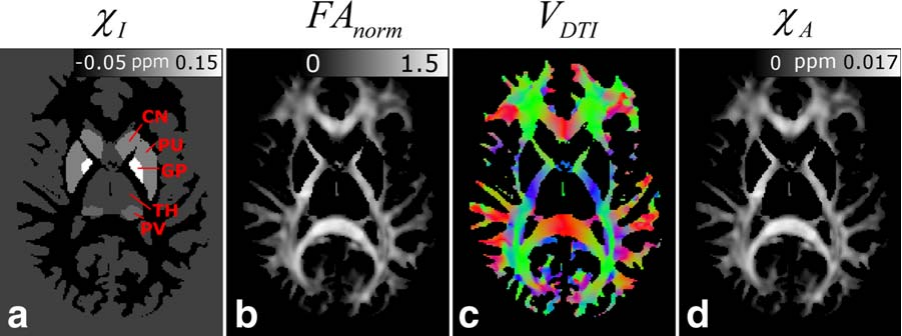
Representative axial slices at the level of the optic radiations of the data sets used to carry out whole brain frequency simulations. (a) Isotropic susceptibility map,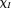, based on a segmented white matter image from a T1-weightted anatomical image. The deep GM regions included in the isotropic model are also labeled: GP = globus pallidus; PU = putamen; CN = caudate nucleus; TH = thalamus; PV = pulvinar part of the thalamus. (b) DTI-based FA map divided by estimated FA in optic nerve (0.59) to yield a normalized FA-map,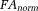, which should be a good indicator of fiber anisotropy. (c) Colour-coded fiber-orientation map (red = left-right, green = anterior–posterior, blue = foot–head) based on the principal eigenvectors (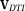) extracted from a DTI dataset. Fiber orientation information was used in calculating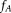 and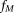. (d) Map of the anisotropic susceptibility, formed by multiplying the normalized FA map (b) by the experimentally measured values of the anisotropic susceptibility of WM in the optic nerve (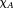 = 0.0113 ppm).

Figure 4 shows representative axial slices at the level of the optic radiations drawn from the data sets that were used in calculating the whole brain frequency maps. Figures 4a and 4d show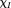 and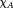 maps, respectively. The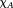-map was formed by multiplying the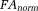 map (Fig. 4b) by the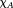-value measured in the optic nerve (0.011 ppm). The principal axis of the susceptibility tensor in each voxel was aligned with the principal axis of the diffusion tensor, defined by unit vector,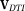 (Fig. 4c).

**Figure 5:**
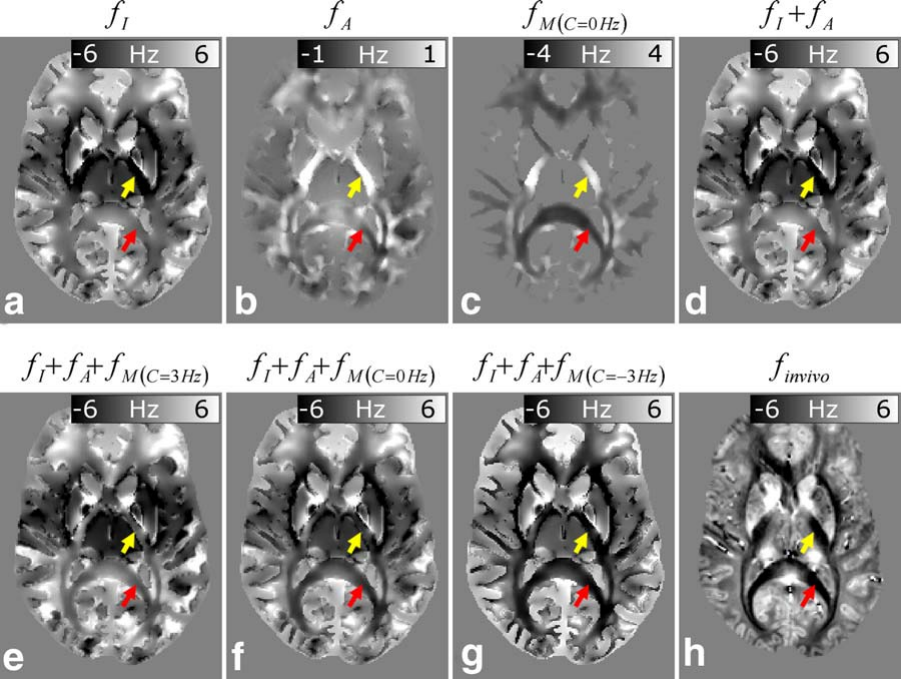
Representative axial slices of simulated frequency data. (a) Simulated frequency map due to the isotropic magnetic susceptibility distribution,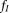. (b) Simulated frequency map due to the anisotropic magnetic susceptibility distribution,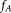. (c) Simulated frequency map due to microstructure,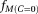. (d) Composite frequency map including frequency shifts due to isotropic and anisotropic magnetic susceptibility,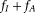. Composite frequency map including frequency shifts due to isotropic and anisotropic magnetic susceptibility, as well as microstructure with three different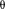-independent offsets (see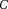 in Eq. [6]): (e) composite frequency map with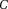 = +3 Hz,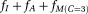; (f) frequency map with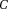 = 0 Hz,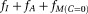; (g) frequency map with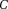 = −3 Hz. For comparison, an in vivo frequency map,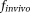, acquired at 7T is also shown (h). The arrows in all images highlight the frequency contrast in the splenium (red arrow), a white matter (WM) region where fibers are oriented perpendicular to *B*_0_, as well as the internal capsule (yellow arrow), a WM region where fibers are oriented parallel to *B*_0_.

Figure 5 shows calculated frequency maps for the same axial slice shown in Figure 4. For comparison, the corresponding slice of the frequency map measured in vivo at 7T,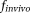, is also displayed (Fig. 5h). The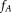 maps (Fig. 5b) show much greater heterogeneity in WM compared with the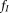 data (Fig. 5a), with offsets that reflect the underlying orientation of the susceptibility tensor (see Fig. 4c). Arrows highlighting the internal capsule (yellow arrow), where the local WM fiber orientation is parallel to *B*_0_, and the splenium (red arrow), where the local WM fiber orientation is perpendicular to *B*_0_, are shown in all images. Comparison of Figures 5a and 5b indicates that the frequency offsets produced by the anisotropic susceptibility (± 1 Hz) are significantly smaller than those produced by the isotropic susceptibility (± 6 Hz). This is a consequence of the relatively small magnitude of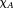, which took a maximum value of 0.017 ppm compared to WM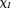-value of −0.05 ppm. The positive isotropic susceptibility of the deep GM structures introduces negative offsets in the surrounding WM in this axial slice through the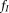-map, with particularly negative offsets associated with the internal capsule region (yellow arrow in Fig. 5a). The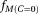 map shows similar heterogeneity in WM to the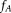 map, but the variations are much larger, with frequency offsets in the range of ± 4 Hz. Figure 5d shows the composite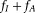 map and Figure 5e to 5g shows composite maps formed by including the microstructure-related frequency offset with three different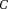-values: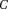 = −3 Hz (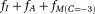);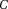 = 0 Hz (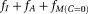);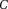 = 3 Hz (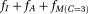). Comparison of these maps with Figure 5h indicates that the composite maps that incorporates the effect of microstructure (Fig. 5e–5g) are generally more similar to the measured frequency map. In particular, they exhibit negative frequency offsets in the splenium and optic radiations that better match the observed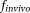 frequency behavior compared to the rather low contrast of the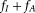 image (see red arrows). Of the three composite maps that include the effect of microstructure, the highest level of agreement between simulations and measured values is observed for the negative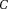-value (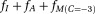 in Fig. 5g).

**Figure 6:**
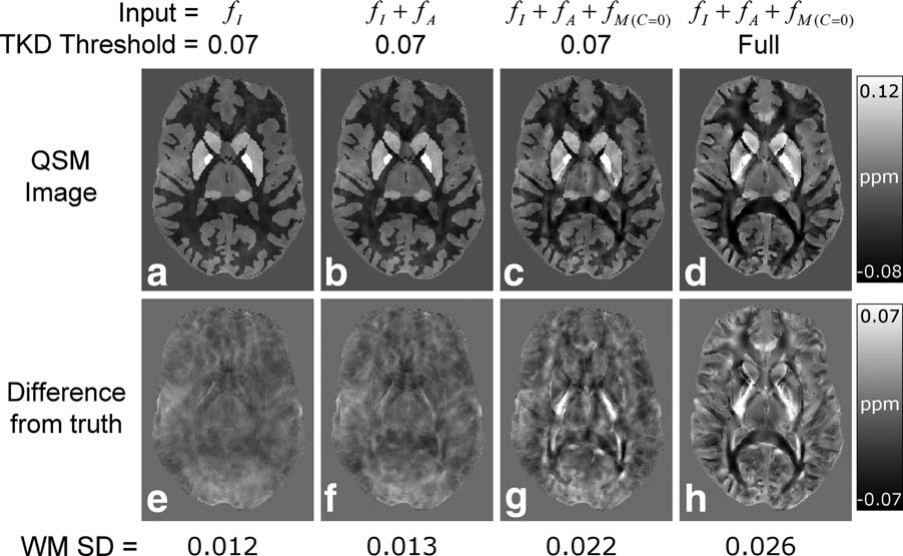
Representative slices showing the results of the QSM calculations. The figure shows susceptibility maps produced by applying TKD-based QSM with a truncation threshold of 0.07 to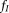 (A),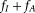 (B), and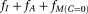 (C) data. The susceptibility map generated by applying the fully relaxed TKD method to the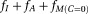 data is also shown (d). Difference from truth (DFT) images, generated by subtracting the isotropic susceptibility map used in the model (see Fig. 4a) from each calculated susceptibility map, are also shown (e–h). The standard deviation of susceptibility values in white matter, which was used in this study to indicate artefactual heterogeneity, is listed below the DFT images.

Figure 6 shows the results of the QSM calculations for the same axial slices as used in Fig. 4 and Fig. 5. The standard deviation (SD) of the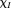-values in WM was calculated for each dataset to provide an indication of the error in the calculated susceptibility maps; and the values are listed in Figure 6. The susceptibility map calculated from the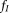-data (Fig. 6a) exhibits fairly homogenous diamagnetic WM contrast (SD = 0.012 ppm), and the associated DFT image (Fig. 6e) shows there are only small differences that result from the replacement of data by constant values in a small region of k*-*space in the TKD method. The susceptibility map calculated from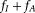 (Fig. 6b) has a similar appearance to the map derived from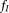 alone, but it exhibits a slightly increased level of artefactual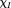-variation in WM (SD = 0.013 ppm). The susceptibility map calculated from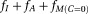 using a TKD threshold of 0.07 (Fig. 6c) shows increased heterogeneity in WM (SD = 0.022 ppm) relative to the maps produced from the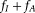 or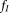 data. The associated DFT image (Fig. 6g) clearly shows that the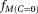 frequency offsets (Fig. 5c) propagate into the final QSM image as artefacts. From a qualitative perspective, the susceptibility map calculated from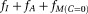 using a fully relaxed TKD kernel (Fig. 6d) appears to show reduced artefact levels and clearer boundaries relative to the map calculated from the same data using a TKD threshold of 0.07 (Fig. 6c). However, this qualitative improvement comes at the cost of a general increase in WM heterogeneity (SD = 0.026 ppm), also clearly shown in the associated DFT image (Fig. 6h). For comparison, the standard deviations of the frequency values in WM for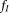,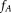, and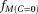 (see Fig. 5) were found to be 3.14 Hz / 0.011 ppm, 0.34 Hz / 0.001 ppm, and 1.02 Hz / 0.003 ppm, respectively.

**Figure 7:**
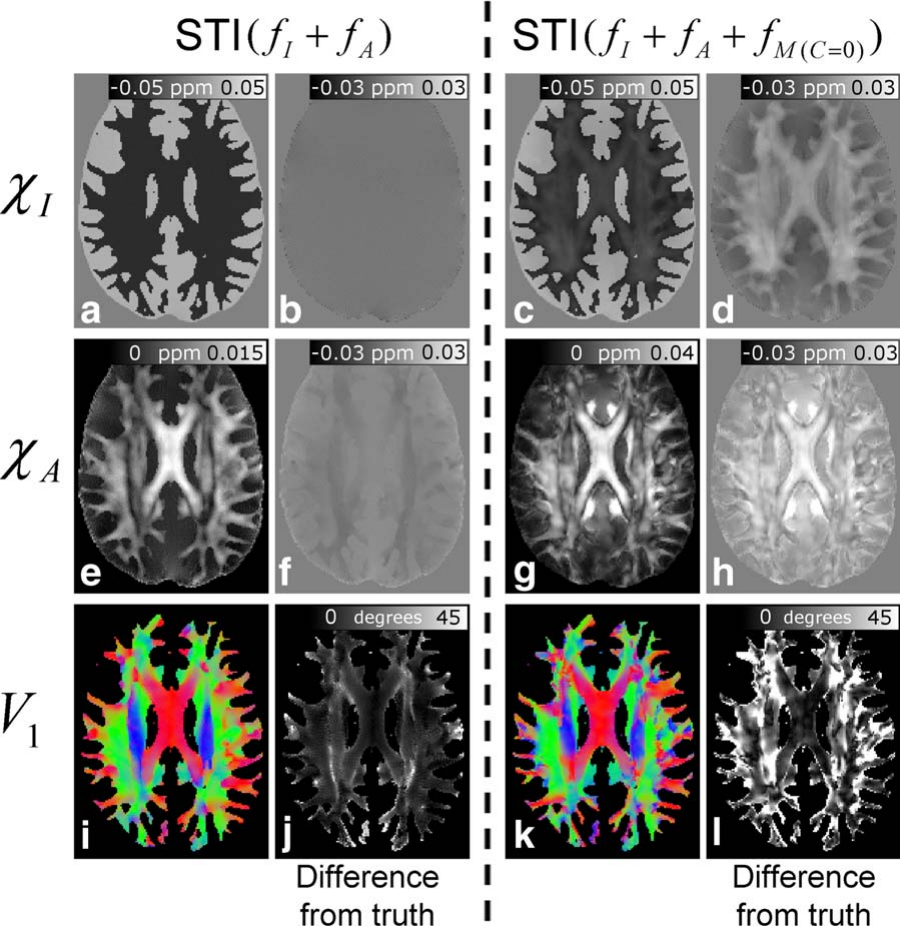
Representative slices showing the results of applying STI to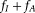 (left hand side) and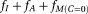 (right-hand side) data. The reconstructed isotropic (A & C) and anisotropic susceptibility maps (E & F) are shown alongside the associated difference from truth images (B, D, F & H). The colour-coded reconstructed fiber orientation maps are shown (I & K) alongside difference from truth images (J & L) which depict the magnitude of the angle between the reconstructed tensor orientation and the orientation used in the model (see Fig. 4c).

Figure 7 shows the results of the investigation of the effect of frequency offsets due to microstructure on STI. The left-hand and right-hand sides of Figure 7 show the results of applying the STI processing to the composite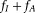 and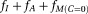 data. The representative axial slices shown here lie at the level of the CC. The reconstructed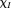 (Fig. 7a),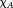 (Fig. 7e), and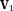 (Fig. 7i) maps calculated from the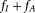 frequency data are generally in excellent agreement with the ground truth maps (Fig. 4), as evidenced by the low associated DFT values (Figs. 7b, 7f, and 7j). Despite this general agreement, some small errors inherent to the STI processing can be seen in the DFT image associated with the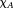-map (Fig. 7f). To probe these discrepancies further, an ROI was drawn in the CC. The average value of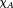 in this region of the model used in the simulations was found to be 0.011 ± 0.003 ppm. Here, the error is the standard deviation over the ROI, which reflects the spatial variation of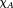 in the model due to variation of the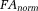 term used in its calculation (see Eq. [6] in simulating whole brain frequency maps in Methods). The average value of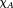 in the CC ROI applied to the map derived from the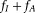 data was 0.013 ± 0.004 ppm, which is a factor of 1.18 times larger than the ground truth. The mean angle between the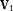 vectors calculated from the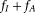 data and the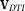 vectors, which defined the direction of the principal axis of the susceptibility tensor in the model data, was 18 ± 17^o^ in WM. In contrast to the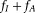 results, the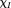,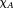, and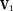 maps calculated from the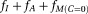 data, which include the effect of microstructure (Fig. 7, right-hand side) show considerable errors when compared to the original model data, as evidenced by the large offsets in the DFT images. In particular, the large positive offsets in the DFT image associated with the reconstructed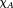 map (Fig. 7h) suggest that the anisotropy in WM is strongly overestimated in these data. This is also apparent from the average value of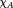 in the CC ROI, which is 0.030 ± 0.010 ppm, that is, a factor of 2.8 times larger than the value in the model data. The generally positive offset in WM regions of the DFT image associated with the reconstructed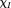 map (Fig. 7d) also suggests that STI based on the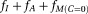 data underestimates the diamagnetic isotropic susceptibility of WM. The mean angle between the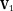 vectors calculated from the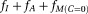 data and the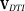 vectors was larger than for the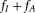 data, taking an average value of 28 ± 23^o^ in WM voxels.

## DISCUSSION

The fitted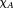 value of 0.011 ppm in the optic nerve experiments corresponds to an overall susceptibility anisotropy of
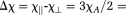
0.017 ppm. This is larger than the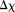 = 0.012 ppm value estimated by Lee et al. 18, who applied a similar approach to a fixed, postmortem human CC sample. The discrepancy could be due to fixation effects because, unlike Lee et al.'s sample, our sample was not fixed; but the discrepancy could also be due to differences in tissue degradation. Our sample was scanned only 4 hours after harvesting, while the time between harvesting and fixation for a human sample is likely to be significantly longer. The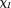 value of −0.082 ppm measured here suggests that WM is diamagnetic relative to agarose gel, and this value is similar to the −0.116 ppm value measured by Luo et al. 22 in a rat optic nerve using a similar approach, but with formalin as the reference medium. These results add to the growing body of evidence suggesting that WM is diamagnetic relative to water. The average residual frequency inside the optic nerve sample varied by 5.59 Hz as the nerve orientation was rotated from parallel to perpendicular to the field, showing a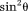 dependence on the angle of the nerve to the field that is consistent with the predicted effect of WM microstructure 16,19,31. Using a forward model that only incorporated bulk isotropic susceptibility, Luo et al. 22 also measured a similar orientation-dependent frequency offset that, after conversion into Hz at 7 T and adopting the sign conventions used in our work, would yield approximate coefficients of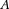 = −6.7 Hz and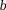 = 4.5 Hz in Eq. [5]. These values are of the same sign and of a similar magnitude to the microstructure-related offsets measured here using a purely isotropic model (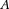 = −6.53,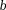 = 5.97 Hz). Discrepancies between the two estimates of *b* may be due to the different choice of reference media. Our reference medium was agarose gel, which may exhibit exchange effects that are not present in the water reference used by Luo et al. 22. However, the results of this study (see Suppl. Fig. 1) strongly suggest that an anisotropic component should be included in forward models used for simulating WM phase contrast.

By considering the simulated frequency maps shown in Figure 5, we can draw several important inferences about the effects of anisotropy and microstructure on phase images acquired in vivo using GE MRI. First, the induced frequency offsets due to the macroscopic distribution of anisotropic magnetic susceptibility (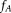
≈ ± 1 Hz in Fig. 5b) are much smaller in magnitude than the frequency contribution due to microstructure (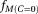
≈ ± 4 Hz in Fig. 5c). Second, inclusion of a microstructure contribution yields simulated frequency maps (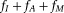 in Fig. 5) that are in better correspondence with in vivo data (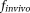 in Fig. 5h) than maps in which this contribution is omitted (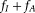 in Fig. 5d). This effect is especially apparent in the splenium (see red arrows in Fig. 5). The deep GM structures induced negative frequency offsets in the internal capsule that appear to act against the strongly positive contrast yielded by the microstructure contribution (see yellow arrows in Fig. 5) to yield image contrast in closer agreement with the in vivo map,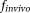. The composite map incorporating microstructure with a negative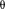-independent frequency offset,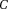 = −3 Hz (Fig. 5g), yielded image contrast in closest agreement with the experimental data. This result suggests that a positive exchange contribution in WM relative to GM, as reported in recent studies 19,21,43, is unlikely to be a dominant source of contrast for in vivo frequency maps at the TE values and field strength used in this study. A generally negative offset is consistent with the predictions of the hollow cylinder fiber model presented in our previous work 19, which suggests that the radial anisotropy of the myelin sheath will induce a negative offset in the axonal compartment that reaches a maximum magnitude when the fiber orientation is perpendicular to *B*_0_. It is likely that the level of agreement between simulated and measured frequency data could be improved upon by using a more sophisticated model of WM. In this work, FA maps are used to indicate the coherence of fiber distributions in WM voxels, but a better approach might be to use diffusion spectroscopy imaging (DSI). Also, information on axonal density and myelin content, which is known to vary over the different WM regions of the brain, could be incorporated into the model. Despite these limitations, the simulations do strongly suggest that microstructure effects must be included in any accurate modeling of frequency contrast in WM. To simplify the QSM and STI experiments, the value of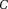 was set equal to zero (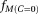). Consequently, further work also needs to be carried out to investigate the artifacts induced in susceptibility maps by non-zero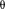-independent WM frequency offsets.

The results of the QSM calculations demonstrate the potentially confounding influence of microstructure, and to a lesser extent anisotropy, on the measurement of isotropic susceptibility values in vivo. The inclusion of frequency contrast due to anisotropy only slightly increased the measured WM heterogeneity in the reconstructed susceptibility map (see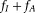 results in Fig. 6) relative to that observed in the susceptibility map based on frequency contrast due to a purely isotropic susceptibility distribution (see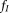 results in Fig. 6). However, the inclusion of the frequency contribution from microstructure caused a dramatic increase in WM heterogeneity in the calculated susceptibility map, as evidenced by the doubling of the measured SD of the WM susceptibility (see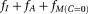 results in Fig. 6). The spatial variation of the induced heterogeneity due to microstructure is closely linked to the underlying fiber orientation. More sophisticated inversion methods that incorporate edge priors and noise reduction strategies 12,46 may reduce this unwanted heterogeneity, but are unlikely to remove it completely. Also, many of the structures exhibiting large microstructure effects, such as the splenium and optic radiation, can appear in the edge-based priors used by these algorithms, and therefore may be particularly difficult to remove. These results suggest that care should be taken when inferring the relative abundance of iron and myelin from QSM measurements in WM.

In contrast to QSM, STI processing appropriately accounts for the frequency variation,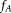, due to voxel-scale anisotropic susceptibility. The accuracy of the STI inversion methodology is clearly demonstrated by the faithful reconstruction of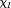,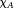, and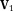 maps from the “ideal”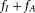 frequency data as shown in Figure 7 (left-hand side). However, the results based on the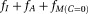 data (Fig. 7, right-hand side) suggest that the inclusion of a frequency contribution from microstructure, which is not accounted for in STI processing, can induce significant errors in the calculated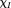,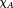, and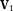 maps. Most striking, inclusion of the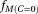 contribution causes significant overestimation of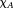-values in all WM regions: In the CC region, the average reconstructed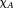 value was measured to be almost a factor of three times larger than the value used in the model. An implication of this result is that much of the anisotropy measured in WM in previous in vivo STI studies could potentially be due to the effect of microstructure.

## CONCLUSION

The effects on QSM and STI of the frequency variation produced by the anisotropic susceptibility and microstructure of WM were investigated in this study. This involved characterizing for the first time the frequency offsets in WM that are not explained by the effect of bulk isotropic or anisotropic magnetic susceptibility, but rather result from the local microstructure. The relative isotropic magnetic susceptibility and absolute anisotropic magnetic susceptibility of a fresh sample of optic nerve were quantified by imaging the sample at multiple orientations to the *B_0_*-field and analyzing the field perturbation produced outside the sample. Residual frequency maps were formed by subtracting simulated frequency maps from the measured frequency values. The local microstructure-related frequency contribution was then characterized by measuring the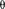-dependence of the average residual frequency offset inside the sample. At 7T and with the particular echo times employed (7 ms and 20 ms), this contribution varied by about 5.6 Hz as the nerve orientation varied from parallel to perpendicular to the field. Simulated frequency maps were then calculated using a digitized whole-brain WM model formed from anatomical data and DTI data acquired from a volunteer. The results of the simulations suggest that the frequency contribution of microstructure is much larger than that due to bulk effects of anisotropic magnetic susceptibility. The application of QSM and STI data processing to the simulated frequency maps showed that microstructure-related offsets yield artifacts in the calculated susceptibility maps. In the QSM processing, the microstructure contribution introduced artificial WM heterogeneity in the reconstructed isotropic susceptibility map. For the STI processing, the microstructure contribution caused the susceptibility anisotropy to be significantly overestimated. These findings indicate that further research should be carried out to reduce the confounding effects of microstructure-related frequency contributions in susceptibility mapping, but also provide further evidence that the effect of microstructure on phase images in itself potentially provides a useful new source of contrast.
