# Supplementary material for: Effects of White Matter Microstructure on Phase and Susceptibility Maps
Source: Magn Reson Med. 2014 Mar 11;73(3):1258–69. doi: 10.1002/mrm.25189 (PMC4359018; doi:10.1002/mrm.25189)
Supplement: Supplementary file 1 [file mrm0073-1258-sd1.doc]

**Supplementary Material**

**
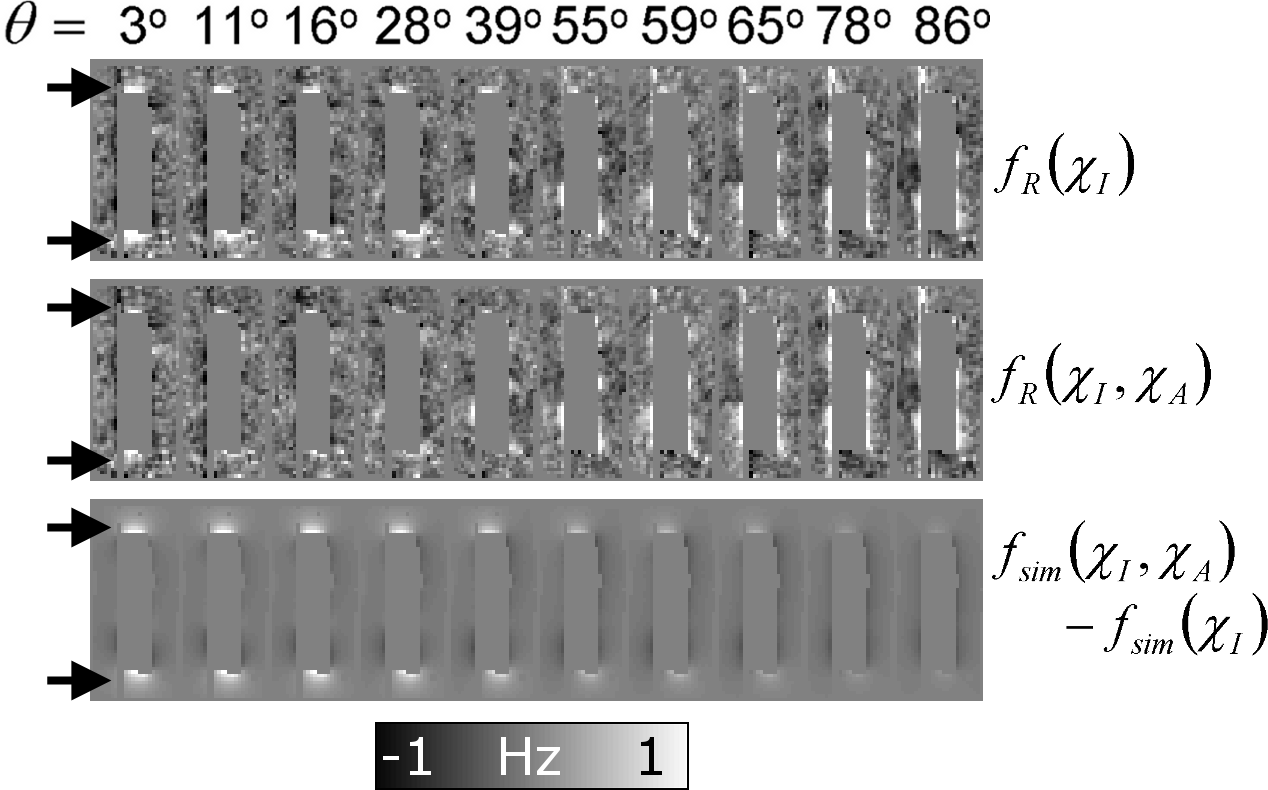
**

**Fig. S1 –** Comparison of the residual frequency maps produced by taking the difference between the experimental data and the best fitting frequency maps produced by a sample with a purely isotropic magnetic susceptibility, $f_{R}\left(\chi_{I}\right)$, and a sample with a combination of isotropic and anisotropic magnetic susceptibility, $f_{R}\left(\chi_{I},\chi_{A}\right)$. The difference between the two models, $f_{sim}\left(\chi_{I},\chi_{A}\right)-f_{sim}\left(\chi_{I}\right)$, is also shown. Only the frequency offsets in external voxels included in the fitting process are shown in all maps. A positive frequency lobe that is on the order of 1 Hz in magnitude can be seen to occur at the top and bottom (see arrows) of the optic nerve sample in the$f_{R}\left(\chi_{I}\right)$-map as $\theta$ approaches 0^o^. This positive residual offset is significantly reduced in the$f_{R}\left(\chi_{I},\chi_{A}\right)$ map by the inclusion of frequency offsets due to anisotropic susceptibility. The $f_{sim}\left(\chi_{I},\chi_{A}\right)-f_{sim}\left(\chi_{I}\right)$ map shows that the inclusion of anisotropic magnetic susceptibility allows the additional positive frequency lobe to be better characterised. These images provide further evidence of the importance of including an anisotropic magnetic susceptibility in forward models used to describe phase contrast in high field MRI.
